# Hospital Burden of All-Cause Pneumonia and Nonbacteremic Pneumococcal Pneumonia in Adults in France Between 2013 and 2019

**DOI:** 10.1093/ofid/ofae349

**Published:** 2024-06-28

**Authors:** Ayman Sabra, Marie Bourgeois, Emmanuelle Blanc, Stephane Fievez, Jennifer Moïsi, Gwenaël Goussiaume, Magali Lemaitre, Laurence Watier, Nicolas Coulombel, Julien Tréhony, Aurore Tricotel, Yasmine Baghdadi, Muriel S Fartoukh

**Affiliations:** Medical Affairs, Vaccines, Antivirals and Evidence Generation, Pfizer, Paris, France; Vaccines Department, Pfizer, Paris, France; Vaccines Department, Pfizer, Paris, France; Health and Value Department, Pfizer, Paris, France; Medical Affairs, Vaccines, Antivirals and Evidence Generation, Pfizer, Paris, France; Vaccines Department, Pfizer, Paris, France; Real World Solutions, IQVIA, Courbevoie, France; Epidemiology and modelling of antibacterial evasion, Institut Pasteur, Paris, France; Real World Solutions, IQVIA, Courbevoie, France; Real World Solutions, IQVIA, Courbevoie, France; Real World Solutions, IQVIA, Courbevoie, France; Real World Solutions, IQVIA, Courbevoie, France; Assistance Publique–Hôpitaux de Paris, Sorbonne Université, Service de médecine intensive réanimation, Hôpital Tenon, Paris, France

**Keywords:** France, mortality, pneumococcal disease, pneumonia, *Streptococcus pneumoniae* vaccination

## Abstract

**Background:**

Community-acquired pneumonia (CAP) is associated with significant morbidity and mortality. The study objective was to describe the hospital burden of pneumonia in the adult population in France.

**Methods:**

This retrospective study was conducted from the National Health Insurance Database. All hospitalizations for pneumonia (all-cause) between 2013 and 2019 were included. Different risk categories for patients were established based on pneumococcal vaccine recommendations by French health authorities.

**Results:**

A total of 2 199 240 episodes of CAP were registered over the study period (annual mean, 314 177 [standard deviation, 17 818.6]); 75% occurred in patients aged ≥65 years, among whom 47% were not classified in the moderate- or high-risk categories recommended for French pneumococcal vaccination. The incidence of CAP increased with age (117.9, 395.3, and 1916.7 per 100 000 for the age groups 18–49, 50–64, and ≥65 years, respectively, in 2019). Furthermore, being at risk of pneumococcal disease resulted in more severe outcomes, including longer episode duration (mean, 14 days in low-risk vs 17 days in high-risk patients) and higher risk of referral to critical care units (from 20% to 27%), of rehospitalization up to 180 days (from 39% to 67%), of in-hospital death (from 12% to 19%), and of 1-year mortality (from 26% to 49%).

**Conclusions:**

This study establishes the incidence of CAP in adults in France, describes the significant burden of disease, and highlights the need for better prevention policies.

## Teaser

This French study was conducted between 2013 and 2019. Among all-cause community-acquired pneumonia hospitalizations, 75% occurred in patients aged ≥65 years, among whom 47% were not classified in the moderate- or high-risk categories recommended for French pneumococcal vaccination.

Community-acquired pneumonia (CAP) is a major cause of morbidity and mortality, especially in older people [1, 2]. *Streptococcus pneumoniae* is responsible for 20%–37% of community-acquired pneumonia (CAP) in Europe, and also causes invasive pneumococcal disease (IPD) [3–9]. As for CAP, age is a major risk factor for pneumococcal disease (PD), with higher incidence in subjects aged ≥65 years [1]. In addition to age, chronic diseases (including but not limited to diabetes, chronic respiratory disease, heart failure, and chronic liver disease) and immunosuppression have also been identified as risk factors for PD [1, 2, 10]. Immunocompromised patients and immunocompetent patients with an underlying disease predisposing to PD are targeted by the French NITAG (National Immunization Technical Advisory Groups) recommendations for pneumococcal vaccination [11]. While cases of IPD are routinely monitored through the national surveillance system [12], there are no recent data describing the burden of episodes of hospitalized CAP and their health outcomes in France. A French study assessed determinants of mortality among pneumococcal CAP patients admitted in the intensive care unit, but did not report disease incidence [13]. For nonbacteremic pneumococcal CAP (NBPP), both disease incidence and outcomes are poorly described, as etiological testing is not systematically performed in hospitalized patients with pneumonia [7] and conventional microbiological tests have poor diagnostic performance [3, 7, 8, 14].

The current study primarily aimed to describe the burden of hospitalized all-cause pneumonia in adults in France, between 2013 and 2019, using the National Health Insurance Database. As a secondary analysis, we compared CAP overall to NBPP. The burden of IPD, including invasive pneumococcal pneumonia (PP), was also investigated.

## METHODS

### Data Source and Design

We conducted a retrospective observational study using the French National Health Database (Système National des Données de Santé [SNDS]) [15]. The SNDS is a claims database including 99% of the population living in France and contains all reimbursed outpatient health expenditures linked to hospital discharge summaries from the national hospital discharge database (PMSI). The PMSI provides information on all stays in public or private hospitals, including information on diagnoses using *International Classification of Diseases* (*ICD-10*) codes. The PMSI database is released on an annual basis with a 1-year lag, and hospital stays are part of the PMSI of the year of discharge.

The study included all adults aged ≥18 years with a diagnosis of pneumonia or IPD at the time of hospital admission. Hospitalizations were identified from the PMSI for Medicine Surgery Obstetrics (acute care; Supplementary Document 1). To describe patients’ characteristics, the SNDS was used.

### Study Episodes Population: Definitions and Identification Process

**Figure 1.**
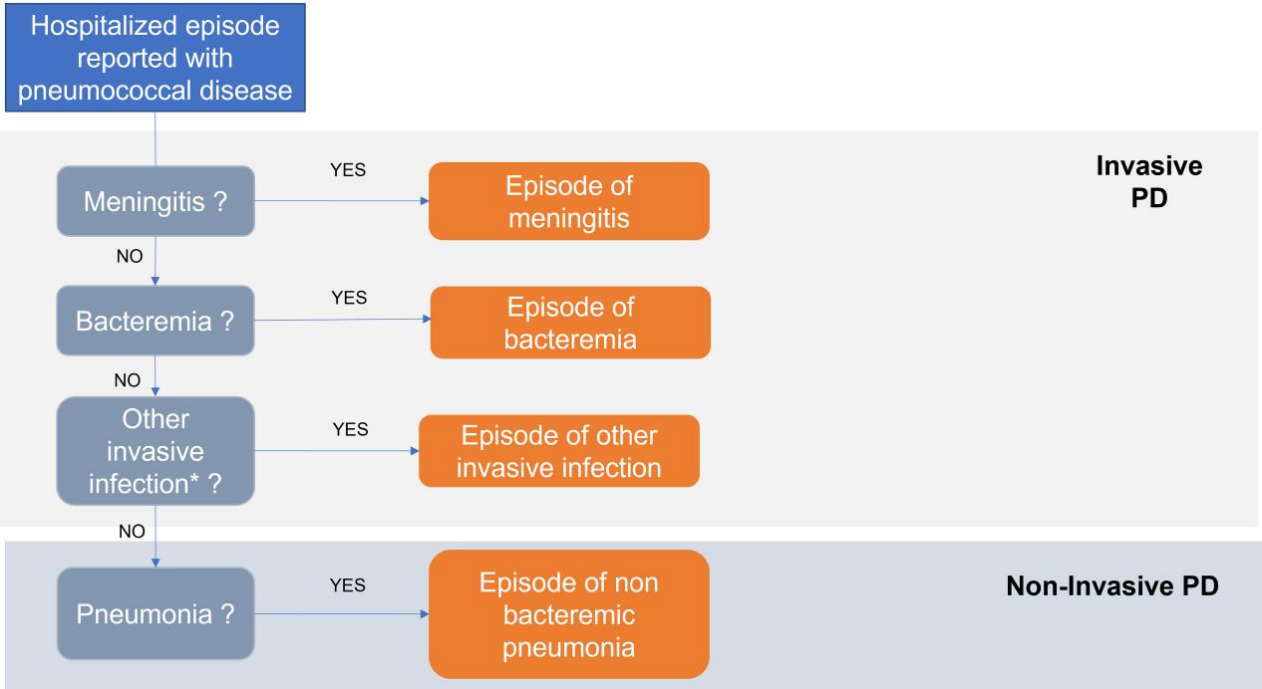
Prioritization of invasive pneumococcal infections. *Other invasive infections: arthritis, osteomyelitis, endocarditis, and nonrheumatic aortic valve disease (further detailed in Supplementary Table 2). Episodes of other infection were excluded from the analysis. Abbreviation: PD, pneumococcal disease.

All hospital stays recorded with a primary diagnosis or a secondary diagnosis (diagnosis in other position than primary) of pneumonia and/or PD with an admission date between 1 January 2013 and 31 December 2019 were selected; those ending in 2020 were not recorded. For identification of PD, unless an *ICD-10* code specific to pneumococcal infection was reported (eg, J13 [pneumonia due to *Streptococcus pneumoniae*], G00.1 [pneumococcal meningitis]), the code B953 (*S pneumoniae* as the cause of diseases classified to other chapters) was also required. Depending on the reported diagnoses, hospital stays were classified into (1) all-cause pneumonia (including bacteremic and NBPP); (2) PP; (3) pneumococcal bacteremia; (4) pneumococcal meningitis; and (5) other IPD (codes detailed in Supplementary Table 1). When multiple diagnoses of PD (PP, bacteremia, meningitis, and other IPD) were recorded within the same stay, the most severe diagnosis was retained with prioritization first given to (1) pneumococcal meningitis; (2) pneumococcal bacteremia, (3) other IPD, and (4) PP. Thus, hospitalizations for NBPP were defined by exclusion of IPD (Figure 1). All PD categories were therefore mutually exclusive.

For patients with multiple stays, we considered 2 distinct episodes if the interval between 2 consecutive stays (from the discharge date of the previous stay to the entry date of the subsequent stay) was >30 days. Otherwise, these stays were considered as a single episode. In that case, the diagnosis of the first stay was always retained, except for a stay of all-cause pneumonia followed within 1 week by a stay for PD. In such case, the diagnosis of the second stay was retained, assuming that the pneumococcal infection was not diagnosed during the first stay but was already present.

### Study Patient Population

A 4-year look-back period was used to categorize patients into 3 risk groups depending on their risk of PD according to risk factors listed by the French High Council for Public Health (HCSP) [11], as follows: (1) high-risk group: immunocompromised patients; (2) moderate-risk group: immunocompetent patients with an underlying disease; and (3) low-risk group (eg, Alzheimer disease or without any comorbidity detailed in Supplementary Document 2 and Supplementary Table 2). Risk groups were identified using algorithms from the COVARISQ (COuverture VAccinale des adultes à RISQues) study monitoring pneumococcal vaccination uptake in France [16] and other publications [17–20] for comorbidities not described in COVARISQ. For incidence rate estimation by risk group, a different approach was used (algorithms described in the HCSP report [21]), as patients who died during the year were not recorded in COVARISQ.

### Variables

Patients’ baseline characteristics were described (age, sex, risk of PD). Episodes were described in terms of length of stay (defined as the cumulative duration of each stay in case of multiple hospital stays), transfer to critical care units (including transfer to intensive care units and affiliated intermediate care wards, and other continuous monitoring units), and discharge modes (in-hospital death and transfer to follow-up and rehabilitation care). Rehospitalizations for respiratory causes (primary diagnosis with *ICD-10* codes J00–J99), cardiac causes (primary diagnosis with *ICD-10* codes I00–I99), or any causes, as well as all-cause deaths, were also recorded after an episode.

### Statistical Analyses

Analyses were conducted at the episode level, per calendar year and, if relevant, over the whole study period. For the estimation of mortality risk, only the first episode of the calendar year was considered. Except for costs and mortality rates, all analyses were stratified by age (18–49, 50–64, ≥65 years) and risk group according to the current local recommendations for pneumococcal vaccination [11].

#### Incidence, Rehospitalization, and All-Cause Mortality Rates

Incidence rates were calculated using the population on 1 January of the given year from the French census as a denominator [22]. An episode was counted for calendar year in which it started. The incidence rates per risk group were estimated using the risk groups defined in the HCSP report, which were only available for 2019 (approach is detailed in Supplementary Documents 2 and 3); thus, results stratified by age group are only presented for 2019.

Independent of monitoring episodes, rehospitalization and mortality risk were assessed between 2013 and 2018, at 30 and 180 days from discharge and at 30, 180, and 365 days from admission date, respectively.

#### Direct Hospital Economic Burden

Direct hospital costs per hospitalized episode were estimated using data from the collective perspective Echelle Nationale Commune des Coûts (ENCC; national cost study), based on hospital accounting data by Diagnosis Related Group (DRG) collected in a nationally representative sample of facilities, as recommended by current French guidelines [23] (see Supplementary Document 3 for further details). For episodes with multiple hospital stays, the cost of an episode was calculated as the sum of the costs of each hospital stay. Each resource consumed was valued in euros over the reference year 2019.

### Patient Consent

This study does not include factors necessitating patient consent.

## RESULTS

### Hospitalization Episodes of Interest

Between 2013 and 2019, a total of 2 199 240 hospitalization episodes of all-cause pneumonia were recorded, of which 70 290 (3.2%) were identified as episodes of NBPP. In addition, there were 21 523 and 3655 hospitalization episodes of pneumococcal bacteremia and pneumococcal meningitis, respectively.

### Characteristics of Patients

On average, the annual number of episodes of all-cause pneumonia was 314 177 (standard deviation [SD], 17 818.6). Men represented 54% and patients aged ≥65 years represented 75% of the total number of episodes. Interestingly, 47% of these episodes occurred in patients considered low risk in the current pneumococcal vaccine recommendations.

Similar trends were observed for episodes of NBPP, with 58% occurring in men, 60% in patients aged ≥65 years, and among them, 48% of patients who were not considered at increased risk of PD.

Irrespective of the age of patients, the distribution of risk factors for PD was roughly similar for all-cause pneumonia and NBPP, with approximately half of patients classified as low-risk (49% and 53%, respectively) (Table 1).

**Table 1.** Description of Hospitalization Episodes of All-Cause Pneumonia and Nonbacteremic Pneumococcal Pneumonia in Terms of Patient Characteristics in France, Annual Average 2013–2019

Characteristic	All-Cause Pneumonia	NBPP
All adults		
Mean No. of episodes (SD)	314 177 (17 818.62)	10 041 (1250.68) (3.2% of all-cause pneumonia)
Sex, male	168 705 (53.7)	5828 (58.0)
Age		
18–49 y	30 637 (9.7)	1587 (15.8)
50–64 y	49 301 (15.7)	2384 (23.7)
≥65 y	234 238 (74.5)	6071 (60.5)
Age, y, median (IQR)		
All adults	78 (64.0–87.0)	69 (57.0–82.0)
Low risk	78 (61.0–87.0)	67 (52–82)
Moderate risk	81 (70.0–87.0)	75 (63.0–84.0)
High risk	67 (57.0–76.0)	64 (54.0–73.0)
Risk group		
Low risk	154 788 (49.3)	5330 (53.1)
Moderate risk	121 764 (38.8)	3573 (35.6)
High risk	37 624 (12.0)	1138 (11.3)
Comorbidities		
Chronic respiratory disease^a^	50 985 (16.2)	1859 (18.5)
Heart failure	60 180 (19.2)	1305 (13.0)
Diabetes	54 351 (17.3)	1504 (15.0)
18–49 y		
Risk group		
Low risk	20 970 (68.4)	1148 (72.4)
Moderate risk	4484 (14.6)	231 (14.5)
High risk	5183 (16.9)	208 (13.1)
Comorbidities		
Chronic respiratory disease^a^	2273 (7.4)	119 (7.5)
Heart failure	833 (2.7)	35 (2.2)
Diabetes	1463 (4.8)	69 (4.3)
50–64 y		
Risk group		
Low risk	23 913 (48.5)	1243 (52.1)
Moderate risk	14 468 (29.3)	757 (31.8)
High risk	10 920 (22.1)	384 (16.1)
Comorbidities		
Chronic respiratory disease^a^	8732 (17.7)	473 (19.8)
Heart failure	3903 (7.9)	151 (6.3)
Diabetes	7333 (14.9)	320 (13.4)
≥65 y		
Risk group		
Low risk	109 906 (46.9)	2939 (48.4)
Moderate risk	102 812 (43.9)	2585 (42.6)
High risk	21 521 (9.2)	547 (9.0)
Comorbidities		
Chronic respiratory disease^a^	39 979 (17.1)	1268 (20.9)
Heart failure	55 444 (23.7)	1119 (18.4)
Diabetes	45 555 (19.4)	1116 (18.4)

Data are presented as No. (%).

Abbreviations: IQR, interquartile range; NBPP, nonbacteremic pneumococcal pneumonia; SD, standard deviation.

^a^Chronic obstructive pulmonary disease, emphysema.

### Incidence Rates of All-Cause Pneumonia

The annual incidence rate of hospitalization episodes of all-cause pneumonia tended to increase (+12.3%) over the period 2013–2019, varying from 577 to 648 episodes per 100 000 persons (/100K) (Table 2).

**Table 2.** Number of Episodes and Incidence Rates (per 100 000 Inhabitants) of All-Cause Pneumonia by Age Group—France, 2013–2019

Year	18–49 y		50–64 y		≥65 y		All Adults	
	No.	IR	No.	IR	No.	IR	No.	IR
2013	31 328	116.7	48 889	385.8	214 229	1859.9	294 446	577.0
2014	30 086	112.6	47 425	372.8	209 348	1763.2	286 859	559.0
2015	30 395	114.4	49 254	386.0	233 588	1911.3	313 237	607.6
2016	32 102	121.5	50 133	392.2	239 056	1904.3	321 291	620.7
2017	29 194	111.1	48 497	378.4	242 785	1885.8	320 476	616.6
2018	31 519	120.2	52 135	406.3	254 155	1931.3	337 809	647.1
2019^a^	30 790	117.9	50 850	395.3	258 041	1916.7	339 681	647.8

Abbreviation: IR, incidence rate.

^a^For the year 2019, numbers of episodes and corresponding incidence rates were adjusted to account for hospitalization episodes ending in 2020.

**Figure 2.**
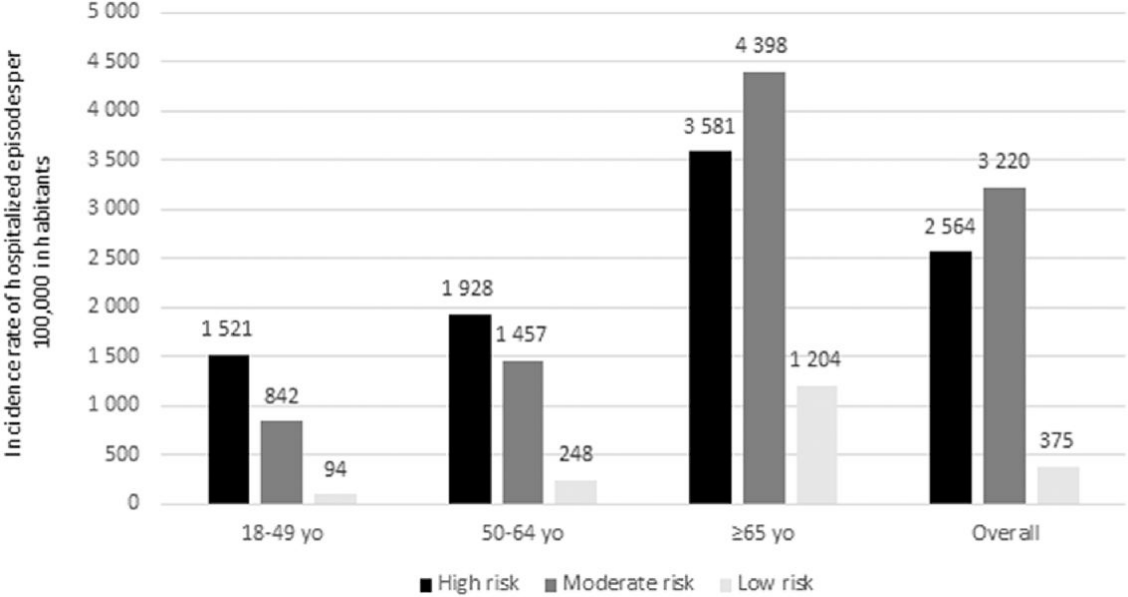
Incidence rate (per 1 000 000 inhabitants) of hospitalization episodes of all-cause pneumonia, by age and risk group in 2019 in France. Incidence rates were adjusted to account for hospitalization episodes ending in 2020.

When focusing on the most recent year, 2019, the incidence of all-cause CAP was not necessarily the highest in the high-risk group. In fact, this was only observed in patients <65 years (1521 and 1928/100K for the age groups 18–49 and 50–64 years, respectively). In patients ≥65 years of age, who represent the majority of cases, the highest reported incidence was in the moderate-risk group (4398/100K) (Figure 2). Information on NBPP, bacteremia, and meningitis is shown in Supplementary Figure 1.

### All-Cause Pneumonia Hospital Outcomes

Over the study period, when considering episodes of all-cause pneumonia, the risk of adverse outcomes including transfer to critical care units, prolonged length of stay, and in-hospital death tended to increase with the PD risk category (Table 3). Mean length of hospital stay ranged from 14 days (median, 10 days) in low-risk patients to 17 days (median, 12 days) in high-risk patients, the risk of transfer to critical care unit increased from 20.3% to 27.0%, and in-hospital death increased from 11.5% to 19.0% (Table 3). Regardless of the risk category, the in-hospital death risk also increased with age (13.5% mortality in the age group ≥65 years for all risk groups). Within each risk group, similar risk of transfer to critical care unit was observed in patients aged 18–49 and 50–64 years (33% and 32%; 39% and 42%; 29% and 33%, respectively, for patients at high, moderate, and low risk of PD), but rates in patients aged ≥65 years were systematically lower at 23%, 20%, and 16%, respectively. Conversely, the risk of transfer to rehabilitation care was higher in patients classified at low or moderate risk of PD and increased with age within each risk category.

**Table 3.** Characteristics of Hospitalization Episodes of All-Cause Pneumonia in Adults by Risk Group and Age Group Over the Whole Study Period in France

Characteristic	18–49 y	50–64 y	≥65 y	Overall Episodes
High risk				
No. of episodes	36 281	76 441	150 647	263 369
Length of stay, d				
Mean (SD)	17.6 (22.62)	18.8 (22.03)	16.5 (16.61)	17.3 (19.24)
Median (IQR)	10 (6.0–20.0)	12 (6.0–23.0)	12 (7.0–21.0)	12 (6.0–21.0)
Transfer in critical care unit^a^, No. (%)	12 060 (33.2)	24 499 (32)	34 601 (23)	71 160 (27.0)
In-hospital death, No. (%)	3460 (9.5)	14 423 (18.9)	32 153 (21.3)	50 036 (19.0)
Transfer to rehabilitation care^b^, No. (%)	1575 (4.9)	5158 (8.6)	15 962 (14.1)	22 695 (11.1)
Moderate risk				
No. of episodes	31 390	101 278	719 681	852 349
Length of stay, d				
Mean (SD)	15.9 (21.05)	17.6 (20.9)	15 (13.93)	15.4 (15.26)
Median (IQR)	9 (5.0–18.0)	11 (6.0–20.0)	11 (7.0–18.0)	11 (7.0–19.0)
Transfer in critical care unit^a^, No. (%)	12 135 (38.7)	42 086 (41.6)	144 386 (20.1)	198 607 (23.3)
In-hospital death, No. (%)	1957 (6.2)	10 858 (10.7)	109 952 (15.3)	122 767 (14.4)
Transfer to rehabilitation care^b^, No. (%)	2149 (7.5)	9362 (10.7)	103 477 (17.9)	114 988 (16.5)
Low risk				
No. of episodes	146 788	167 393	769 341	1 083 522
Length of stay, d				
Mean (SD)	12.6 (18.88)	15.7 (20.12)	14.2 (13.6)	14.2 (15.59)
Median (IQR)	7 (4.0–13.0)	9 (5.0–18.0)	11 (7.0–17.0)	10 (6.0–17.0)
Transfer in critical care unit^a^, No. (%)	42 352 (28.9)	55 458 (33.1)	122 496 (15.9)	220 306 (20.3)
In-hospital death, No. (%)	4749 (3.2)	13 337 (8.0)	106 680 (13.9)	124 766 (11.5)
Transfer to rehabilitation care^b^, No. (%)	9183 (6.5)	14 961 (9.8)	123 932 (19.2)	148 076 (15.8)
All episodes				
No. of episodes	214 459	345 112	1 639 669	2 199 240
Length of stay, d				
Mean (SD)	13.9 (19.99)	17.0 (20.82)	14.8 (14.06)	15 (15.98)
Median (IQR)	8 (4.0–15.0)	10 (6.0–20.0)	11 (7.0–18.0)	11 (6.0–18.0)
Transfer in critical care unit^a^, No. (%)	66 547 (31.0)	122 043 (35.4)	301 483 (18.4)	490 073 (22.3)
In-hospital death, No. (%)	10 166 (4.7)	38 618 (11.2)	248 785 (15.2)	297 569 (13.5)
Transfer to rehabilitation care^b^, No. (%)	12 907 (6.4)	29 481 (9.8)	243 371 (18.2)	285 759 (15.5)

Abbreviations: IQR, interquartile range (Q1–Q3); SD, standard deviation.

^a^Transfer in critical care unit includes transfer to resuscitation unit, intensive care unit, and continuous monitoring unit.

^b^Among patients still alive at hospital discharge.

Mortality was 14% within 30 days following hospital admission, 27% within 180 days, and 33% within 365 days (Supplementary Table 3). Mortality rates increased with the risk of PD, at 12%, 15%, and 19% at 30 days in patients at low, moderate, or high risk of PD, respectively; 22%, 29%, and 41% at 180 days; and 26%, 36%, and 49% at 365 days.

Among patients who survived their first episode of CAP, 18% were rehospitalized within 30 days of discharge and 48% within 180 days. We also explored the contribution of cardiac and respiratory events to rehospitalization; these rates were 3% within 30 days and 11% within 180 days for cardiac causes and 3% and 12%, respectively, for respiratory causes (Supplementary Table 4). Again, the risk of all-cause rehospitalization at 30 and 180 days increased with PD risk level (Supplementary Table 4). Rates of rehospitalization for respiratory causes were higher among patients identified at risk of PD (15%–16% vs 8% in patients of the low-risk group) at 180 days, with no effect of age, but fairly similar at 30 days (3% vs 2% in patients of the low-risk group). Rehospitalizations for cardiac causes increased with age and were higher in patients from the moderate-risk group compared to patients from other risk groups (5% vs 3% and 2% at 30 days and 16% vs 9% and 7% at 180 days in high- and low-risk patients, respectively).

### Direct Hospital Cost of Episodes of All-Cause Pneumonia

The mean annual total direct cost of all episodes of all-cause pneumonia was 2490.47 million euros, with 69% of these costs attributable to patients aged ≥65 years (results not shown).

In 2019, mean hospital cost per episode of all-cause pneumonia increased with risk category from 6892 to 8237 euros in patients classified at low risk and at high risk of PD, respectively (Supplementary Table 5).

### Episodes of NBPP Versus Episodes of All-Cause Pneumonia

Compared to episodes of all-cause pneumonia, whatever the risk group, episodes identified as NBPP were at higher risk of transfer to critical care unit (38% vs 22%) but had similar rates of in-hospital death (11.2% vs 13.5%), transfer to rehabilitation care (14% vs 16%), and length of stay (median, 11 days) (Supplementary Table 6).

In terms of rehospitalization (all-cause, cardiac, or respiratory causes), the same trends as for all-cause pneumonia were observed for episodes identified as NBPP, but rates were slightly lower (Supplementary Table 7). Mortality rates were also lower in patients with NBPP (24% at 365 days vs 33% in patients with all-cause pneumonia) (Supplementary Table 8). In contrast, mean costs per episode were higher for episodes of NBPP compared to episodes of all-cause pneumonia and similar across risk categories, from 8194 to 8505 euros in 2019 (Supplementary Table 9).

Further results, including data on the burden of IPD, will be described in a future publication.

## DISCUSSION

### Main Results

This study aimed to update the epidemiologic and economic burden of hospitalization episodes of all-cause pneumonia in France.

Overall, patients aged ≥65 years accounted for 75% of episodes. In this population, 1 of 2 patients was not eligible for pneumococcal vaccination according to current French recommendations. Indeed, in France vaccination is only recommended for immunocompromised patients and nonimmunocompromised patients with an underlying disease predisposing to PD. In 2018, 4.5% of patients at risk were up to date with their pneumococcal vaccination (a 13-valent pneumococcal conjugate vaccine followed by a 23-valent pneumococcal polysaccharide vaccine) [24].

Interestingly, the “low-risk” population, which is currently not considered a target for vaccination by health authorities, accounted for almost half the cases reported in this study with an in-hospital mortality rate of 11.5% and a rehospitalization rate of 8.5% (for respiratory causes) within 6 months of discharge. In addition, the economic burden was also high in all low-risk patients with mean costs per episode amounting to 6892 euros, versus 7433 and 8237 euros in patients classified as moderate and high risk of PD. These data altogether show that there is a gap in the current recommendation and that a considerable burden of disease could still be averted if vaccine recommendations were to be widened to all adults aged ≥65 years.

Among patients aged ≥65 years, a higher incidence of all-cause pneumonia in the moderate-risk group than in the high-risk group was reported. This could be explained by the increased incidence rates in people with concurrent at-risk conditions [1].

Recent estimates of incidence of hospitalizations from all-cause pneumonia have been lacking in France so far. Several European studies conducted over a similar period provide context for our results. In an Italian study conducted from the hospital discharge database of the Italian Health Ministry, the incidence rate of all-cause pneumonia was estimated as 496/100K in 2019 [25]. However, a higher incidence was reported in Germany in a study conducted on representative healthcare claims database [26] (1054 per 100K adult population in 2015 vs 608/100K in the current study). For comparison, in the United States, for example, incidence of CAP in adults was roughly similar to that reported here (649/100K) [27].

While a disparity at the national level cannot be ruled out, these differences observed between countries might be explained by differences in case definitions. Thus, the higher incidence observed in the German study could be related to the broader case definition used, with 3 additional *ICD-10* codes (B01.2 [varicella pneumonia], J69.0 [respiratory disease due to solid and liquid substances], and J85.1 [lung abscess with pneumopathy not due to microorganism]). These codes, and particularly J69, are not related to infectious etiology and may lead to an overestimation of the burden of all-cause pneumonia. Another contributor could be difference in vaccination recommendations and uptake across countries [24, 28–30].

### Costs

In our study, in 2019, mean cost per episode of all-cause pneumonia was €7334 (SD, €8002.3). A French study PNEUMOCOST, on hospitalization of confirmed PP between 2011 and 2014, reported an average cost of stay of €7293 (SD, €7363) [31], similar to our results.

### Proportion of NBPP Episodes Among All-Cause Pneumonia Episodes

In the United Kingdom, Pick et al reported that 36.6% of pneumonia was due to *S pneumoniae* in a prospective cohort study [5]. Similarly, in 2013, a meta-analysis estimated the proportion of CAP attributable to pneumococcus at 27.3% (95% confidence interval, 23.9%–31.1%) [3]. Unlike in these studies, where pneumonia etiology was actively investigated and included microbiologically confirmed invasive and nonbacteremic pneumonia, our study relied on standard-of-care testing and coding only to identify pneumococcal cases, and as a result, NBPP only represented 3.2% of all-cause pneumonia. A similarly low proportion was also reported in Italy, with PP representing 2.2% of all-cause pneumonia. These results highlight the lack of systematic etiological testing in clinical practice, especially in case of uncomplicated pneumonia, as well as likely underreporting of pneumonia etiology in the PMSI database. Given the significant underestimation of the proportion of NBPP among CAP, likely affecting the generalizability of results to all NBPP episodes, we focused on all-cause pneumonia and reported NBPP data only in the Supplementary Materials. For example, NBPP was found to have a lower mortality but a higher rate of transfer to critical care unit than all-cause pneumonia, but the imbalance between the number of reported and expected NBPP episodes precludes drawing robust inferences from these observations.

### Strengths and Limitations

Besides the use of the SNDS [32], which covers approximately 99% of French residents over the study period, one of the main strengths of the study is the identification of patients listed at risk of PD according to the HCSP recommendations, allowing us to rigorously describe the incidence and economic burden of all-cause pneumonia (including NBPP) by risk group based on published algorithms [16, 21].

The use of secondary data had also some limitations since data were initially collected for reimbursement purposes and hospitals activities measurements and not for epidemiological or clinical purposes. Notably, results of biological tests or medical procedures are not available, so infectious episodes of interest could only be identified from hospital discharge diagnoses. Consequently, a great majority of pneumonia episodes were reported as unspecified. However, algorithms we used have been defined with clinicians and epidemiologists, who considered them as most accurate and relevant given the available data in the SNDS.

In addition, the method used to estimate costs from the French collective perspective presents limitations intrinsic to the database. Indeed, to approximate cost of hospital stays, we used the recommended source (ENCC, based on samples published for the years 2012 to 2018), in which economic data are based on accounting agreements of a sample of French hospitals [23]. The main drawback in using ENCC is that nonstandard stays and outliers (mostly the costliest) are excluded, thus having a direct impact on mean costs [23]. Moreover, because ENCC costs are directly attached to DRG, collective costs are by nature highly homogeneous. This leads to situations where 2 pathologies partially attached to the same DRG have identical first and second (median) quartile costs. This issue is, however, bypassed using medians to describe costs. Last, functional decline in the elderly was not assessed in this study; however, it has been previously reported that pneumonia negatively impacts activities of daily living and quality-adjusted life-years, as well as extending hospital stays in this population [33, 34].

## CONCLUSIONS

To our knowledge, this is the first study in recent years reporting incidence of all-cause pneumonia in the French hospitalized adult population as well as describing the heavy burden this disease constitutes. We interestingly found that 75% of episodes of all-cause pneumonia occurred in patients 65 years and older, half of whom are not even covered by the current vaccine recommendations against PD, even though *S pneumoniae* is known to be a major contributor of CAP in those patients. Altogether, these findings illustrate the need to take additional prevention measures and, as with influenza vaccination, to extend the current pneumococcal vaccination recommendations to all individuals 65 years and older.

## Supplementary Material

ofae349_Supplementary_Data

## Supplementary Data

Supplementary materials are available at *Open Forum Infectious Diseases* online. Consisting of data provided by the authors to benefit the reader, the posted materials are not copyedited and are the sole responsibility of the authors, so questions or comments should be addressed to the corresponding author.

## Notes

***Acknowledgments.*** We thank our Pfizer colleagues Christian Theilacker and Maria Lahuerta for their critical review of the manuscript.

***Disclaimer.*** All authors contributed to the design of the study. N. C. and J. T. accessed and analyzed data. A. S., M. B., E. B., S. F., L. W., M. L., N. C., J. T., A. T., Y. B., and M. S. F. interpreted data. Y. B. and A. T. wrote the original manuscript. All authors read and approved the final manuscript.

***Financial support*.** This work was supported by Pfizer. This study was conducted as a collaboration between Muriel Fartoukh, Laurence Watier, IQVIA, and Pfizer.
