# Supplementary material for: Hospital Burden of All-Cause Pneumonia and Nonbacteremic Pneumococcal Pneumonia in Adults in France Between 2013 and 2019
Source: Open Forum Infect Dis. 2024 Jun 28;11(7):ofae349. doi: 10.1093/ofid/ofae349 (PMC11237635; doi:10.1093/ofid/ofae349)
Supplement: ofae349_Supplementary_Data [file ofae349_supplementary_data.docx]

**Supplementary materials**

# Supplementary document 1: Description of health data system

The National hospital discharge database (PMSI, *Programme de Médicalisation des Systèmes d’Information*) database covers all hospital stays in French publicly funded and private hospitals. It is updated annually and used for reimbursement purposes. Therefore, each hospitalization record is standardized, following the Technical Agency for Hospital Information (ATIH, *Agence technique de l’information sur l’hospitalisation*) recommendations. It contains hospital diagnoses and medical procedures performed during each stay. Diagnoses are coded as principal diagnosis (PD: condition requiring hospitalization), related diagnosis (RD: adds information to PD) and significant associated diagnosis (SAD: complications and co-morbidities potentially affecting the course or cost of hospitalization).

# Supplementary document 2: Approach used to estimate incidence rate of hospitalized episode by risk group

According to the high council of public health (HCSP) risk groups of pneumococcal diseases were defined as follow [1]:

- - **Immunocompromised patients (High-risk category)**: asplenia or hyposplenia, hereditary immune deficits, HIV, chemotherapy-treated cancer, solid organ transplant, hematopoietic stem cell transplant, chronic autoimmune or inflammatory diseases treated by immunosuppressive or biologic drugs, nephrotic syndrome.
  - **Non-immunocompromised patients with an underlying disease predisposing to pneumococcal invasive infections (Moderate-risk category):** chronic respiratory disease (COPD, emphysema), severe asthma under continuous treatment, cyanotic heart disease, heart failure, end-stage renal failure with replacement therapy, dialysis, renal transplant, post-transplant follow up, chronic liver disease, diabetes, osteomeningeal breach, cochlear implant.
  - **Patients not classified at risk of Pneumococcal Diseases (Low-risk category):** sleep apnea, tobacco use, alcohol use, morbid obesity, malnutrition, Alzheimer disease, Parkinson disease, multiple sclerosis, patients with no comorbidities.

The incidences rates per risk group were estimated using the risk groups defined in the report for the French High Council for Public Health (HCSP) [2], only available for 2019. This is the reason why results are only presented for this year. At-risk population in the study was defined as follows:

1/ Prevalence of each risk factors of interest was estimated based on the methodology developed in a report for HCSP. The proposed method mainly used data of the French National Health Insurance (NIH) [3] on the number of persons in France with coverage for a Long-Term Disease (LTD) of comorbidities of interest. For 4 risk factors not part of the report for HCSP (hematopoietic stem cell transplant, chronic autoimmune or inflammatory diseases treated by immunosuppressive or biologic drugs, osteo-meningeal breach or cochlear implants) results from the COVARISQ study were used [4].

For some risk factors, corrective factors needed to be applied to rectify under- or over-estimation of prevalence of the disease. All diabetic patients are not covered for LTD, a corrective factor (1.29), identified from a report from NIH, was applied to rectify underestimation of the diabetic population in France [5]. Moreover, since patients with a cancer under chemotherapy cannot be flagged through the cancer LTD, which considers all patients regardless of a treatment, a corrective factor (15.15%), derived from data of the National institute of cancer, was applied to rectify overestimation [6].

2/ Population at risk in each risk group – High-, Moderate-, and Low-risk of PD – was estimated applying the same algorithm as per the denominators in the study population. As patients could have at least two risk factors from the same risk group (e.g., diabetes and renal failure), corrective factors were applied to manage duplicates within risk groups. The factors applied were determined from the COVARISQ study [4].

3/ Incidence rate of the four pathologies of interest (all-cause pneumonia, NBPP, pneumococcal bacteremia and meningitis) by risk group in 2019, in France was estimated as study population at risk divided by prevalence of risk group per 100 000 inhabitants.

**References**

1. Haut Conseil de Santé Publique (HCSP). Avis relatif aux recommandations vaccinales contre les infections à pneumocoque pour les adultes [Internet]. 2017 Oct. Available from: https://www.hcsp.fr/Explore.cgi/Telecharger?NomFichier=hcspa20170310_infectpneumocoquerecomadultes.pdf

2. Beutels P, Blommaert A, Willem L, Lepoutre A, Lévy-Bruhl D. Etude médico-économique de vaccins: Vaccination antipneumococcique des adultes. 2017.

3. Assurance Maladie. Améliorer la qualité du système de danté et maîtriser les dépenses [Internet]. Available from: https://assurance-maladie.ameli.fr/sites/default/files/2021-07_rapport-propositions-pour-2022_assurance-maladie_0.pdf

4. Wyplosz B, Fernandes J, Goussiaume G, Moïsi J, Lortet-Tieulent J, Vainchtock A, et al. Adults at risk of pneumococcal disease in France. Infectious Diseases Now. 2021 Nov;51(8):661–6.

5. Assurance maladie. Dépenses remboursées affectées à chaque pathologie en 2019 | L’Assurance Maladie [Internet]. [cited 2022 Oct 21]. Available from: https://assurance-maladie.ameli.fr/sites/default/files/2021-07_rapport-propositions-pour-2022_assurance-maladie_3.pdf

6. L’ACTIVITÉ EN CANCÉROLOGIE POUR LA CHIMIOTHÉRAPIE [Internet]. Available from: https://www.e-cancer.fr/Professionnels-de-sante/Les-traitements/Chimiotherapie/Chiffres-cles-de-la-chimiotherapie

# Supplementary document 3: Additional methods specification

*Incidence, rehospitalization and all-cause mortality rates*

An episode was counted for calendar year in which it started. For example, an episode starting in November 2016 and ending in January 2017 (‘overlapping episode’) was counted for the year 2016. To take into account overlapping episodes between 2019 and 2020 in the calculation of the incidence for 2019, 2019 incidence rates by age group were adjusted using the mean proportion of overlapping episodes among all stays between 2013 and 2018.

*Direct hospital economic burden*

Direct hospital costs per hospitalized episode were estimated using data from the collective perspective Echelle Nationale Commune des Coûts (ENCC; national cost study), based on hospital accounting data by Diagnosis Related Group (DRG) collected in a nationally representative sample of facilities, as recommended by current French guidelines [1]. The collective perspective includes costs reimbursed by national health insurance (health insurance perspective), medical professionals costs and running costs of hospital stays, complementary insurance and remaining out of pocket charges.

1. HAS / Service évaluation économique et santé publique. Choix méthodologiques pour l’évaluation économique à la HAS. 2020 p. 118. Available at: https://www.has-sante.fr/upload/docs/application/pdf/202007/guide_methodologique_evaluation_economique_has_2020_vf.pdf. Accessed 30 November 2023.

# Table S1 ICD-10 codes used to identify episodes of all-cause pneumonia or pneumococcal infection

| **Label** | **ICD-10 codes**  **[Primary, or secondary diagnoses]** |
| --- | --- |
| **All-cause pneumonia** | |
| Legionnaires disease | A481 |
| Influenza with pneumonia, seasonal influenza virus identified | J100 |
| Influenza with pneumonia, virus not identified | J110 |
| Viral pneumonia, not elsewhere classified | J12* |
| Pneumonia due to *Streptococcus pneumoniae* | J13 |
| Pneumonia due to *Haemophilus influenzae* | J14 |
| Pneumonia due to *Klebsiella pneumoniae* | J15.0 |
| Pneumonia due to *Pseudomonas* | J15.1 |
| Pneumonia due to *Staphylococcus* | J15.2 |
| Pneumonia due to S*treptococcus*, group B | J15.3 |
| Pneumonia due to other streptococci | J15.4 |
| Pneumonia due to other Gram-negative bacteria | J15.6 |
| Pneumonia due to *Mycoplasma* pneumoniae | J15.7 |
| Other bacterial pneumonia | J15.8 |
| Bacterial pneumonia, unspecified | J15.9 |
| Pneumonia due to other specified infectious organisms | J16.8 |
| Pneumonia in bacterial diseases classified elsewhere | J17.0 |
| Pneumonia in viral diseases classified elsewhere | J17.1 |
| Pneumonia in mycoses | J17.2 |
| Pneumonia in other diseases classified elsewhere | J17.8 |
| Pneumonia, organism unspecified | J18* |
| **Pneumococcal Disease** | |
| **Pneumococcal pneumonia (including non-bacteremic pneumococcal pneumonia)** | |
| Pneumonia due to *Streptococcus pneumoniae* | J13 |
| Algorithm based on combination of:   - diagnosis codes related to all-cause pneumonia - evidence of Streptococcus pneumoniae [ICD 10 code B953 - *Streptococcus pneumoniae* as the cause of diseases classified to other chapters] | (A481 OR J100 OR J110 OR J12*-J18*)  AND  B953 |
| **Pneumococcal bacteremia** | |
| Sepsis due to *Streptococcus pneumoniae* | [A403](https://www.aideaucodage.fr/cim-a403) |
| Algorithm based on combination of:   - diagnosis codes related to bacteremia/sepsis [ICD 10 codes A40 – *Streptococcus pneumoniae* sepsis, A41 - Other sepsis, R572 - Septic shock, R650 - Systemic Inflammatory Response Syndrome of infectious origin without organ failure, R651 - Systemic Inflammatory Response Syndrome of infectious origin with organ failure] - evidence of *Streptococcus pneumoniae* [ICD 10 code B953] | (A40*-A41* OR R572 OR R650 OR R651)  AND  B953 |
| **Pneumococcal meningitis** | |
| Pneumococcal meningitis | [G001](https://www.aideaucodage.fr/cim-g001) |
| Algorithm based on combination of:   - diagnosis codes related to meningitis [ICD 10 codes G00 - Bacterial meningitis, not elsewhere classified, G001 - Meningitis in bacterial diseases classified elsewhere, G002 - Meningitis in other infectious and parasitic diseases classified elsewhere] - evidence of *Streptococcus pneumoniae* [ICD 10 code B953] | (G00*-G02*)  AND  B953 |
| ***Other invasive pneumococcal infections*** | |
| Pneumococcal arthritis and polyarthritis | [M001](https://www.aideaucodage.fr/cim-m001) |
| Algorithm based on combination of:   - diagnosis codes related to symptoms or pathologies compatible with an invasive infection, selected by the scientific committee [ICD 10 codes M00 - Pyogenic arthritis, M01 - Direct infections of joint in infectious and parasitic diseases classified elsewhere, I33 - Acute and subacute endocarditis, I35 - Non-rheumatic aortic valve disease, M86 - Osteomyelitis] - evidence of *Streptococcus pneumoniae* [ICD 10 code B953] | (M00*-M01* OR I33* OR I35* OR M86*)  AND  B953 |

# Table S2: Algorithms for the identification of risk factors and other comorbidities of interest

| Disease | Algorithm | Source | LTD† | Hospitalization (ICD10 and CCAM†† codes) | | | Drugs or medical device | Lab tests |
| --- | --- | --- | --- | --- | --- | --- | --- | --- |
|  |  |  | ICD-10 | ICD-10 | Diagnoses | Procedures | Codes | Codes |
| ***Immunocompromised patients (high risk)*** | | | | | | | | |
| Asplenia or hyposplenia | LTD and/or Hospitalizations | New algorithm | D730 | C261, D561, D570, D572, D730-D735, D738, D739, P151, Q890, S3600, S3601, Z8502 | PD / RD / SAD ‡ | FFFA001, FFFA002, FFFC001, FFFC420, FFQX005, HEPA004, HEPA007, HNFA004, HNFA006, HNFA010, HNFA013, HNFC002, HNQX007 |  |  |
| Hereditary Immune deficits | LTD and/or Hospitalizations | New algorithm | D81-D89, G113 | D81-89, G11.3 | PD / RD / SAD |  |  |  |
| HIV | LTD and/or Hospitalizations and/or Drugs and/or Biologic tests | National Health Insurance [1] | B20*, B21*, B22*, B23*, B24*, Z21 | B20*, B21*, B22*, B23*, B24*, Z21, F024, Z206 R75, O987 | MCO: PD / RD  PSY: PD / AD |  | ≥3 reverse, protease transcriptase inhibitors and HIV antivirals in a year | 0805, 0806, 1691 4117, 4122 |
|  |  |  |  |  | PD / RD (*RUM*) or SAD |  |  |  |
| Chemotherapy-treated cancer | Hospitalizations and/or drugs | New algorithm |  | Z511, Z08.2 | PD / RD |  | ≥3 oral anticancer drugs in a year |  |
| Solid organ transplant | LTD and/or Hospitalizations and/or Drugs | New algorithm | LTD28 | Z940-944, Z9481, Z9482 | PD / RD / SAD | JAEA003, JAEA002, DZEA002, DZEA003, DZEA001, DZEA004, GFEA005, GFEA002, GFEA003, GFEA007, GFEA004, GFEA006, HGEA002, HGEA004, HLEA001, HNEA900, HNEA002, HNEH900 | ≥3 anti-rejection drugs in a year |  |
| Hematopoietic stem cell transplant | Hospitalizations | New algorithm |  | Z948*, (PD/RD/SAD) DRG: 27Z02*, 27Z04J | PD / RD / SAD | FELF010, FELF009 |  |  |
| Chronic autoimmune or inflammatory diseases treated by immunosuppressive or biologic drugs | [LTD and/or Hospitalizations] and Drugs | National Health Insurance [1]  + specific drugs | K50*, K51*, L93*, L94*, M05*, M06*, M45*, M46*, M074, M075, M30*, M08, M09, M31*, M32*, M33*, M34*, M35*, M36 | K50*, K51*, L93*, L94*, M05*, M06*, M45*, M46*, M074, M075, M30*, M08, M09, M31*, M32*, M33*, M34*, M35*, M36 | PD / RD |  | ≥3 immunosuppressive or biologic drugs in a year |  |
|  |  |  |  |  | SAD |  |  |  |
| Nephrotic Syndrome | LTD and/or Hospitalizations | New algorithm | N04* | N04* | PD / RD / SAD |  |  |  |
| ***Non-immunocompromised patients with an underlying disease predisposing to pneumococcal invasive infections (moderate risk)*** | | | | | | | | |
| Chronic respiratory disease (COPD, emphysema) | LTD and/or Hospitalizations | New algorithm | J42*, J43*, J44*, J60*, J61*, J63*, J64*, J65*, J66*, J67*, J684, J701, J702, J703, J82*, J84*, J961* | J42*, J43*, J44*, J60*, J61*, J63*, J64*, J65*, J66*, J67*, J684, J701, J702, J703, J82*, J84*, J961* (PD/RD/SAD) | PD / RD |  |  |  |
|  |  |  |  |  | SAD |  |  |  |
| Severe asthma under continuous treatment | [LTD and/or Hospitalizations] and Drugs | Bouée et al. 2018 [2] | [J45*, J46 (Asthma) AND ≥10 inhaled corticoid + long acting beta-2] AND NOT J40*, J41*, J42* (COPD) | idem LTD | J45, J46: PD / RD  J40, J41, J42: PD / RD / SAD |  | Omalizumab at year n  or ≥10 inhaled (corticoid + long-acting beta-2) |  |
| Cyanotic heart disease | Hospitalizations | New algorithm |  | Q20*-Q25* | PD / RD / SAD |  |  |  |
| Heart failure (HF) | LTD and/or Hospitalizations | National Health Insurance | I50*, I11*, I13* | HF: I50* | PD (RUM) or RD |  |  |  |
|  |  |  |  | Complications: I110, I130, I132, I139, K761*, J81 | PD (RUM) with  DR / SAD of HF |  |  |  |
|  |  |  |  | HF: I50* | SAD, or RD (RUM) |  |  |  |
| End-stage renal failure with replacement therapy |  |  |  |  |  |  |  |  |
| - Dialysis | Hospitalizations and/or *Forfaits* | National Health Insurance |  | DRG:  11K021, 11K022, 11K023, 11K024, 11K02J, 28Z01Z, 28Z02Z, 28Z03Z, 28Z04Z, 28Z05Z, 28Z06Z |  | JVJB001, VJF004, VJF008, JVRP004, JVRP007, JVRP008, YYY007 |  | Prestation codes** : 2129, 2131, 2132, 2134, 2135, 2136, 2137, 2138, 2139, 2140, 2142, 2143, 2144, 2145, 2146, 2334 |
| - Renal transplant | Hospitalizations |  |  | Z940  DRG: 27C06Z, 27C061, 27C062,  27C063, 27C064,  24M39Z, 11M171,  11M172, 11M173,  11M174 | PD / RD / SAD | JAEA003, HNEA002 |  |  |
| - Post transplant follow up | Drugs AND [hospitalization or LTD] |  |  |  |  |  | ATC codes: L04AA06, L04AA10, L04AA18, L04AD01, L04AD02, L04AX01 |  |
| Chronic liver disease | LTD and/or Hospitalizations and/or Drugs | National Health Insurance [1] | B18*, I85*, K70*, K71*, K72*, K73*, K74* | B18*, I85*, K70*, K71*, K72*, K73*, K74* | PD / RD |  | ≥ 3 hepatitis C and/or B treatments |  |
|  |  |  |  |  | SAD |  |  |  |
| Diabetes | LTD and/or Hospitalizations and/or Drugs | National Health Insurance [1] | E10*, E11*, E12*, E13*, E14* | E10*, E11*, E12*, E13*, E14* | PD / RD |  | ≥3 (2 if large packaging) diabetes treatments dispensing in the year |  |
|  |  |  |  | Diabetes complications:  G590, G630, G730, G990, H280, H360, I790, L97, M142*, M146*, N083* | PD / RD with diabetes code PD / RD (RUM) or SAD |  |  |  |
| Osteomeningeal breach | Hospitalizations | New algorithm |  | G9780, G9781 | PD / RD / SAD | Procedures related to fistula closure of cerebrospinal fluid |  |  |
| Cochlear implant | Hospitalizations or device | New algorithm |  | Hospitalization with medical device implantation or Z962 | PD / RD / SAD |  |  |  |
| ***Other comorbidities of interest (other risks)****** | | | | | | | | |
| Sleep apnea | Hospitalizations or device | New algorithm |  | G473 (sleep apnea) | PD / RD / SAD | GLMP001, LBLD017 | ≥2 Mandibular advancement splint (MAS) and/or continuous positive airway pressure (CPAP): LPP codes |  |
| Tobacco use | Hospitalizations and/or *prestations* and/or drugs | Bouillon et al., 2015 [3] |  | F17,  Z716,  Z720 | PD / RD / SAD |  | ≥3 drugs used in nicotine dependence: N07BA*, N06AX12 | *prestation* codes**: 9566, 9526, 9527 |
| Alcohol use | LTD and/or hospitalizations | Bouillon et al., 2015 [3] | F10, K70, Z50.2, Z71.4, Z72.1 | F10, G31.2, G62.1, G72.1, E24.4, K86.0, T51, Z71.4, I42.6, K29.2, K70, Z50.2, Z72.1, K85.2 | PD / RD / SAD |  | ≥3 drug deliveries of: N07BB01, N07BB02, N07BB03, N07BB04, N07BB05 |  |
| Morbid obesity | Hospitalizations and/or drugs | ANSM report 2018 [4] |  | E66, T85.50 | PD / RD / SAD | Procedures related to bariatric surgery | ≥3 drug deliveries of A08AB01 |  |
| Malnutrition | Hospitalizations and/or drug | New algorithm |  | E40, E41, E42, E43, E44, E45, E46 | PD / RD / SAD |  | ≥3 deliveries of oral nutritional supplements (LPP codes) |  |
| Alzheimer disease | LTD and/or Hospitalizations and/or Drugs | National Health Insurance [1] | F00, G30 | F00, G30 | PD / RD |  | ≥3 Alzheimer’s drugs: N06DA*, N06DX01 |  |
|  |  |  |  |  | SAD |  |  |  |
| Parkinson disease | LTD and/or Hospitalizations and/or Drugs | National Health Insurance [1] | G20, F023 | G20, F023 | PD / RD |  | ≥3 Parkinson’s drugs |  |
|  |  |  |  |  | SAD |  |  |  |
| Multiple sclerosis | LTD and/or Hospitalizations and/or Drugs | National Health Insurance [1] | G35 | G35 | PD / RD |  | ≥3 anti-multiple sclerosis drugs: L03AB07, L03AB08, L03AB13, L03AX13, L04AA23, L04AA27, L04AA31, N07XX07, N07XX09 |  |

† LTD = Long term disease

†† CCAM = Common classification of medical procedures (Classification Commune des Actes Médicaux)

‡ PD/RD/SAD: Principal Diagnosis/Related Diagnosis/Significant Associated Diagnosis

** outpatient healthcare provision

**** Sleep apnea, tabacco used, alcohol used, morbid obesity and malnutrition can be defined with other codes in other publications.

**References**

1. Caisse Nationale d’Assurance Maladie (CNAM), Direction de la Stratégie, des Etudes et des Statistiques. Méthodologie médicale de la cartographie des pathologies et des dépenses, version G6 (années 2012 à 2017). ameli.fr 2019. https://www.ameli.fr/l-assurance-maladie/statistiques-et-publications/etudes-en-sante-publique/cartographie-des-pathologies-et-des-depenses/methode.php (accessed August 31, 2020).
2. Bouée S, Laurendeau C, Chouaid C, Bourdin A, Ait-Yahia M, Ostinelli J, et al. Identification et estimation de la prévalence de l’asthme sévère en France via l’échantillon généraliste de bénéficiaires. Rev D’Épidémiologie Santé Publique 2018; 66:S205. https://doi.org/10.1016/j.respe.2018.04.034.
3. Bouillon K, Bertrand M, Maura G, Blotière PO, Ricordeau P, Zureik M. Risk of bleeding and arterial thromboembolism in patients with non-valvular atrial fibrillation either maintained on a vitamin K antagonist or switched to a non-vitamin K-antagonist oral anticoagulant: a retrospective, matched-cohort study. Lancet Haematol. 2015 Apr;2(4):e150-9.
4. Rapport ANSM : Utilisation des inhibiteurs de la pompe à protons. Étude observationnelle à partir des données du SNDS, France, 2015.

**Table S3: Mortality after hospitalized episode of all-cause pneumonia overall and by risk-groups, in adults between 2013-2018 in France**

|  | High risk | Moderate risk | Low risk | Overall |
| --- | --- | --- | --- | --- |
| Mean annual number of episodes between 2013 and 2018* | 33,250 | 110,188 | 150,380 | 293,817 |
| Mean annual number of deaths (N) and mortality rate** (%) |  |  |  |  |
| At 30 days | 6,333 (19.0) | 16,521 (15.0) | 18,132 (12.1) | 40,986 (13.9) |
| At 180 days | 13,553 (40.8) | 31,900 (29.0) | 32,725 (21.8) | 78,178 (26.6) |
| At 365 days | 16,384 (49.3) | 39,881 (36.2) | 39,400 (26.2) | 95,665 (32.6) |

*Mean annual number of episodes, only the first episodes of the calendar were considered.

**Mortality rate estimated from admission date, all patients considered. When applicable, only the first episodes of the calendar year were considered for estimation of mortality rate.

**Table S4: Proportion of re-hospitalization after hospitalized episode of all-cause pneumonia** **overall and by age and risk groups in adults between 2013- 2018 in France**

|  | **18-49 yo** | **50-64 yo** | **≥65 yo** | **Overall episodes** |
| --- | --- | --- | --- | --- |
| **Hospitalization rate, n (%) ^£^** | **High risk** | | | |
| Number of episodes | 28,229 | 52,906 | 98,482 | 179,617 |
| At 30 days - all causes | 9,553 (33.8) | 18,828 (35.6) | 29,372 (29.8) | 57,753 (32.2) |
| At 180 days - all causes | 19,442 (68.9) | 36,895 (69.7) | 64,391 (65.4) | 120,728 (67.2) |
| At 30 days - cardiac causes* | 427 (1.5) | 1,232 (2.3) | 3,157 (3.2) | 4,816 (2.7) |
| At 180 days - cardiac causes* | 1,474 (5.2) | 4,201 (7.9) | 10,506 (10.7) | 16,181 (9) |
| At 30 days – respiratory causes** | 772 (2.7) | 1,902 (3.6) | 2,914 (3) | 5,588 (3.1) |
| At 180 days -respiratory causes** | 3,885 (13.8) | 8,514 (16.1) | 14,610 (14.8) | 27,009 (15) |
|  | **Moderate risk** | | | |
| Number of episodes | 25,276 | 77,861 | 517,828 | 620,965 |
| At 30 days - all causes | 5,375 (21.3) | 16,586 (21.3) | 97,776 (18.9) | 119,737 (19.3) |
| At 180 days - all causes | 15,307 (60.6) | 43,589 (56) | 274,288 (53) | 333,184 (53.7) |
| At 30 days - cardiac causes* | 557 (2.2) | 2,969 (3.8) | 26,617 (5.1) | 30,143 (4.9) |
| At 180 days - cardiac causes* | 1,805 (7.1) | 9,489 (12.2) | 88,208 (17) | 99,502 (16) |
| At 30 days – respiratory causes** | 829 (3.3) | 3,190 (4.1) | 17,331 (3.3) | 21,350 (3.4) |
| At 180 days -respiratory causes** | 4,762 (18.8) | 12,992 (16.7) | 80,173 (15.5) | 97,927 (15.8) |
|  | **Low risk** | | | |
| Number of episodes | 12,2288 | 132,035 | 564,900 | 819,223 |
| At 30 days - all causes | 12,875 (10.5) | 19,791 (15) | 79,809 (14.1) | 112,475 (13.7) |
| At 180 days - all causes | 37,208 (30.4) | 51,456 (39) | 228,072 (40.4) | 316,736 (38.7) |
| At 30 days - cardiac causes* | 1,123 (0.9) | 2,542 (1.9) | 15,490 (2.7) | 19,155 (2.3) |
| At 180 days - cardiac causes* | 3,102 (2.5) | 7,524 (5.7) | 50,297 (8.9) | 60,923 (7.4) |
| At 30 days – respiratory causes** | 1,710 (1.4) | 2,463 (1.9) | 10,478 (1.9) | 14,651 (1.8) |
| At 180 days -respiratory causes** | 6,656 (5.4) | 9,637 (7.3) | 48,005 (8.5) | 64,298 (7.8) |
|  | **All episodes** | | | |
| Number of episodes | 175,793 | 262,802 | 1,181,210 | 1,619,805 |
| At 30 days - all causes | 27,803 (15.8) | 55,205 (21) | 206,957 (17.5) | 289,965 (17.9) |
| At 180 days - all causes | 71,957 (40.9) | 131,940 (50.2) | 566,751 (48.0) | 770,648 (47.6) |
| At 30 days - cardiac causes* | 2,107 (1.2) | 6,743 (2.6) | 45,264 (3.8) | 54,114 (3.3) |
| At 180 days - cardiac causes* | 6,381 (3.6) | 21,214 (8.1) | 149,011 (12.6) | 176,606 (10.9) |
| At 30 days – respiratory causes** | 3,311 (1.9) | 7,555 (2.9) | 30,723 (2.6) | 41,589 (2.6) |
| At 180 days -respiratory cause** | 15,303 (8.7) | 31,143 (11.9) | 142,788 (12.1) | 189,234 (11.7) |

^£^Among patients still alive at hospital discharge
**^*^**Episode with a primary diagnosis of cardiac causes (ICD10 codes I00-I99)
^**^Episode with a primary diagnosis of respiratory causes (ICD10 codes J00-J99)

# Table S5: Distribution of direct hospital costs per episodes of all-cause pneumonia in adults in 2019, in France - Collective perspective

|  | **High risk** | **Moderate risk** | **Low risk** | **All episodes** |
| --- | --- | --- | --- | --- |
| Mean (SD) cost per episode (€) | 8,236.5 (8,577.5) | 7,432.6 (7,885.1) | 6,892.2 (7,766.3) | 7,334.0 (8,002.3) |
| Median (Q1-Q3) cost per episode (€) | 5,378 (3,483-10,175) | 5,378 (3,483-8,329) | 5,378 (3,483-7,039) | 5,378 (3,483-8,042) |

Each resource consumed is valued for France, in euros over a reference year, the year 2019

# Table S6: Characteristics of hospitalized episodes of non-bacteremic pneumococcal pneumonia in adults by risk and age groups on the whole study period in France

|  | **Non-bacteremic pneumococcal pneumonia** | | | | | | |
| --- | --- | --- | --- | --- | --- | --- | --- |
|  | **18-49 yo** | **50-64 yo** | | **≥65 yo** | | **Overall episodes** | |
|  | **High risk** | | | | | | |
| **Number of episodes** | 1,453 | 2,685 | | 3,830 | | 7,968 | |
| **Length of stay (days)** |  |  | |  | |  | |
| Mean (SD) | 16 (21.36) | 17.1 (18.84) | | 16.4 (15.78) | | 16.6 (17.96) | |
| Median (Q1 - Q3) | 9 (6.0-17.0) | 11 (7.0-20.0) | | 12 (7.0-20.0) | | 11.0 (7.0-20.0) | |
| **Transfer in critical care unit, N (%)** | 542 (37.3) | 1,081 (40.3) | | 1,278 (33.4) | | 2,901 (36.4) | |
| **In hospital death** | 88 (6.1) | 331 (12.3) | | 611 (16) | | 1,030 (12.9) | |
| **Transfer to rehabilitation care*, N (%)** | 71 (5.3) | 183 (8) | | 382 (12.3) | | 636 (9.4) | |
|  | **Moderate risk** | | | | | | |
| **Number of episodes** | 1,615 | 5,300 | | 18,095 | | 25,010 | |
| **Length of stay (days)** |  |  | |  | |  | |
| Mean (SD) | 19.1 (21.19) | 18.8 (20.34) | | 16.4 (14.59) | | 17.1 (16.48) | |
| Median (Q1 - Q3) | 12 (7.0-23.0) | 13 (8.0-23.0) | | 12 (8.0-20.0) | | 12.0 (8.0-20.0) | |
| **Transfer in critical care unit, N (%)** | 997 (61.7) | 2,952 (55.7) | | 6,096 (33.7) | | 10,045 (40.2) | |
| **In hospital death** | 121 (7.5) | 536 (10.1) | | 2,466 (13.6) | | 3,123 (12.5) | |
| **Transfer to rehabilitation care*, N (%)** | 121 (8.2) | 527 (11.4) | | 2,556 (17) | | 3,204 (15.2) | |
|  | **Low risk** | | | | | | |
| **Number of episodes** | 8,039 | 8,701 | | 20,572 | | 37,312 | |
| **Length of stay (days)** |  |  | |  | |  | |
| Mean (SD) | 14.5 (19.11) | 17.8 (20.66) | | 15.5 (14.87) | | 15.8 (17.36) | |
| Median (Q1 - Q3) | 8 (5.0-16.0) | 11 (7.0-20.0) | | 11 (7.0-18.0) | | 11.0 (7.0-18.0) | |
| **Transfer in critical care unit, N (%)** | 3,617 (45) | 4,164 (47.9) | | 5,829 (28.3) | | 13,610 (36.5) | |
| **In hospital death** | 313 (3.9) | 778 (8.9) | | 2,663 (12.9) | | 3,754 (10.1) | |
| **Transfer to rehabilitation care*, N (%)** | 583 (7.6) | 845 (10.7) | | 3,134 (17.8) | | 4,562 (13.7) | |
|  | **Overall episodes whatever the level of risk** | | | | | | |
| **Number of episodes** | 11,107 | | 16,686 | | 42,497 | | 70,290 |
| **Length of stay (days)** |  | |  | |  | |  |
| Mean (SD) | 15.4 (19.79) | | 18.0 (20.28) | | 16.0 (14.84) | | 16.4 (17.13) |
| Median (Q1 - Q3) | 9.0 (5.0-17.0) | | 12.0 (7.0-21.0) | | 12.0 (8.0-19.0) | | 11.0 (7.0-19.0) |
| **Transfer in critical care unit, N (%)** | 5,156 (46.4) | | 8,197 (49,1) | | 13,203 (31.1) | | 26,556 (37.8) |
| **In hospital death** | 522 (4.7) | | 1,645 (9.9) | | 5,740 (13.5) | | 7,907 (11.2) |
| **Transfer to rehabilitation care*, N (%)** | 775 (7.4) | | 1,555 (10.5) | | 6,072 (17.0) | | 8,402 (13.8) |

*Among patients still alive at discharge and without rehabilitation care before episode

# Table S7: Re-hospitalization after hospitalized episode of non-bacteremic pneumococcal pneumonia, overall and by age and risk group, in adults between 2013- 2018 in France

|  | **18-49 yo** | **50-64 yo** | **≥65 yo** | **Overall** |
| --- | --- | --- | --- | --- |
| **Hospitalization rate, n (%) ^£^** |  |  |  |  |
|  | **High risk** | | | |
| Number of episodes | 1,176 | 1,991 | 2,624 | 5,791 |
| At 30 days - all causes | 300 (25.5) | 546 (27.4) | 677 (25.8) | 1,523 (26.3) |
| At 180 days - all causes | 678 (57.7) | 1,298 (65.2) | 1,638 (62.4) | 3,614 (62.4) |
| At 30 days - cardiac causes* | ≤10 | 46 (2.3) | 66 (2.5) | 119 (2.1) |
| At 180 days - cardiac causes* | 35 (3) | 160 (8) | 254 (9.7) | 449 (7.8) |
| At 30 days – respiratory causes** | 23 (2) | 53 (2.7) | 70 (2.7) | 146 (2.5) |
| At 180 days -respiratory causes** | 153 (13) | 328 (16.5) | 433 (16.5) | 914 (15.8) |
|  | **Moderate risk** | | | |
| Number of episodes | 1,284 | 4,038 | 13,045 | 18,367 |
| At 30 days - all causes | 221 (17.2) | 688 (17) | 2,106 (16.1) | 3,015 (16.4) |
| At 180 days - all causes | 647 (50.4) | 2,070 (51.3) | 6,637 (50.9) | 9,354 (50.9) |
| At 30 days - cardiac causes* | 23 (1.8) | 98 (2.4) | 481 (3.7) | 602 (3.3) |
| At 180 days - cardiac causes* | 64 (5) | 364 (9) | 1,713 (13.1) | 2,141 (11.7) |
| At 30 days – respiratory causes** | 37 (2.9) | 149 (3.7) | 472 (3.6) | 658 (3.6) |
| At 180 days -respiratory causes** | 187 (14.6) | 718 (17.8) | 2,284 (17.5) | 3,189 (17.4) |
|  | **Low risk** | | | |
| Number of episodes | 6,654 | 6,686 | 14,935 | 28,275 |
| At 30 days - all causes | 522 (7.8) | 830 (12.4) | 1,713 (11.5) | 3,065 (10.8) |
| At 180 days - all causes | 1,755 (26.4) | 2,364 (35.4) | 5,439 (36.4) | 9,558 (33.8) |
| At 30 days - cardiac causes* | 47 (0.7) | 100 (1.5) | 283 (1.9) | 430 (1.5) |
| At 180 days - cardiac causes* | 137 (2.1) | 316 (4.7) | 1,036 (6.9) | 1,489 (5.3) |
| At 30 days – respiratory causes** | 78 (1.2) | 121 (1.8) | 276 (1.8) | 475 (1.7) |
| At 180 days -respiratory causes** | 284 (4.3) | 495 (7.4) | 1,276 (8.5) | 2,055 (7.3) |
|  | **Overall episodes** | | | |
| Number of episodes | 9,114 | 12,715 | 30,604 | 52,433 |
| At 30 days - all causes | 1,043 (11.4) | 2,064 (16.2) | 4,496 (14.7) | 7,603 (14.5) |
| At 180 days - all causes | 3,080 (33.8) | 5,732 (45.1) | 13,714 (44.8) | 22,526 (43) |
| At 30 days - cardiac causes* | 77 (0.8) | 244 (1.9) | 830 (2.7) | 1,151 (2.2) |
| At 180 days - cardiac causes* | 236 (2.6) | 840 (6.6) | 3,003 (9.8) | 4,079 (7.8) |
| At 30 days – respiratory causes** | 138 (1.5) | 323 (2.5) | 818 (2.7) | 1279 (2.4) |
| At 180 days -respiratory causes** | 624 (6.8) | 1,541 (12.1) | 3,993 (13) | 6,158 (11.7) |

^£^Among patients still alive at hospital discharge
**^*^**Episode with a primary diagnosis of cardiac causes (ICD10 codes I00-I99)
^**^Episode with a primary diagnosis of respiratory causes (ICD10 codes J00-J99),

# Table S8: Mortality after hospitalized episode of non-bacteremic pneumococcal pneumonia, pneumococcal bacteremia and pneumococcal meningitis, overall and by risk group, in adults between 2013-2018 in France

|  | Non-bacteremic pneumococcal pneumonia | | | |
| --- | --- | --- | --- | --- |
|  | **High risk** | **Moderate risk** | **Low risk** | **Overall episodes** |
| Mean annual number of episodes between 2013 and 2018* | 1,087 | 3,455 | 5,217 | 9,759 |
| Mean annual number of deaths (N) and mortality rate** (%) |  |  |  |  |
| At 30 days | 137 (12.6) | 412 (11.9) | 490 (9.4) | 1,038 (10.6) |
| At 180 days | 305 (28.0) | 783 (22.7) | 829 (15.9) | 1,918 (19.6) |
| At 365 days | 393 (36.2) | 993 (28.7) | 997 (19.1) | 2,383 (24.4) |
|  | **Pneumococcal bacteremia** | | | |
|  | **High risk** | **Moderate risk** | **Low risk** | **Overall episodes** |
| Mean annual number of episodes between 2013 and 2018* | 446 | 1,059 | 1,461 | 2966 |
| Mean annual number of deaths (N) and mortality rate** (%) |  |  |  |  |
| At 30 days | 90 (20.2) | 204 (19.3) | 207 (14.2) | 501 (16.9) |
| At 180 days | 175 (39.3) | 327 (30.9) | 306 (20.9) | 809 (27.3) |
| At 365 days | 217 (48.5) | 393 (37.1) | 343 (23.5) | 953 (32.1) |
|  | **Pneumococcal meningitis** | | | |
|  | **High risk** | **Moderate risk** | **Low risk** | **Overall episodes** |
| Mean annual number of episodes between 2013 and 2018* | 47 | 98 | 373 | 518 |
| Mean annual number of deaths (N) and mortality rate** (%) |  |  |  |  |
| At 30 days | 9 (19.9) | 27 (27.8) | 59 (15.8) | 95 (18.4) |
| At 180 days | 14 (30.2) | 33 (33.7) | 71 (19.1) | 119 (22.9) |
| At 365 days | 17 (35.2) | 35 (35.8) | 75 (20.0) | 126 (24.3) |

*Mean annual number of episodes, only the first episodes of the calendar were considered.

**Mortality rate estimated from admission date, all patients considered. When applicable, only the first episodes of the calendar year were considered for estimation of mortality rate.

# Table S9: Distribution of direct hospital costs per episodes of non-bacteremic pneumococcal pneumonia, pneumococcal bacteremia and meningitis in adults in 2019, in France - Collective perspective

|  | **Non-bacteremic pneumococcal pneumonia** | | | |
| --- | --- | --- | --- | --- |
|  | **High risk** | **Moderate risk** | **Low risk** | **Overall episodes** |
| Mean (SD) cost per episode (€) | 8,505.2 (7,745.6) | 8,342.7 (7,524.8) | 8,194.0 (8,886.1) | 8,295.7 (8,323.9) |
| Median (Q1-Q3) cost per episode (€) | 5,378 (3,483-11,251) | 5,378 (3,736-10,533) | 5,378 (3,483-9,667) | 5,378 (3,483-10,533) |
|  | **Pneumococcal bacteremia** | | | |
|  | **High risk** | **Moderate risk** | **Low risk** | **Overall episodes** |
| Mean (SD) cost per episode (€) | 12,689.2 (9,730.6) | 12,085.5 (9,210.8) | 11,998.7 (9026.2) | 12,190.2 (9,251.0) |
| Median (Q1-Q3) cost per episode (€) | 11,251 (7,256-13,861) | 11,251 (6,625-12,923) | 11,251 (5,825-12,820) | 11,251 (6,242-12,923) |
|  | **Pneumococcal meningitis** | | | |
|  | **High risk** | **Moderate risk** | **Low risk** | **Overall episodes** |
| Mean (SD) cost per episode (€) | 16,153.1 (13,679.0) | 18,220.3 (13,424.5) | 16,560.5 (12633.0) | 16,770.9 (12935.3) |
| Median (Q1-Q3) cost per episode (€) | 10,106 (6,825-25,571) | 16,546 (10,106-25,571) | 10,324 (10,106-25,571) | 10,533 (10,106-25,571) |

| **Incidence rate of hospitalized episodes per 100,000 inhabitants** | 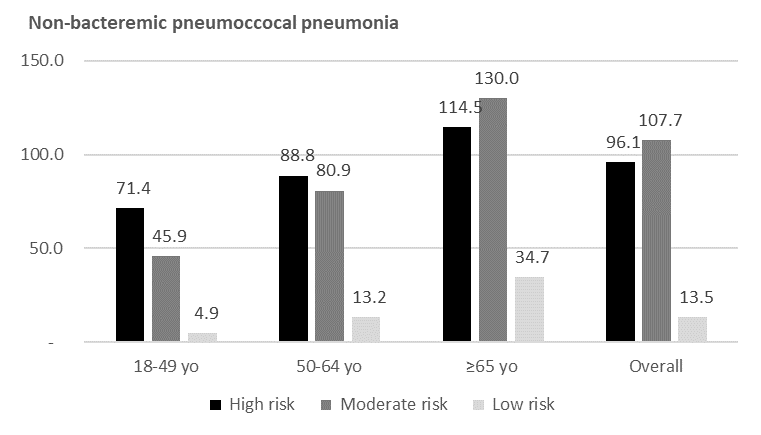 |
| --- | --- |
| **Incidence rate of hospitalized episodes per 100,000 inhabitants** |  |
| **Incidence rate of hospitalized episodes per 100,000 inhabitants** |  |

Figure S1: Incidence rate (per 100,0000 inhabitants) of non-bacteremic pneumococcal pneumonia, pneumococcal bacteremia and meningitis episodes, by age group and risk group in 2019** in France

* Results not presented, numbers ≤10

** Incidence rates were adjusted to account for hospitalized episodes ending in 2020
